# How to Refine and Prioritize Key Performance Indicators for Digital Health Interventions: Tutorial on Using Consensus Methodology to Enable Meaningful Evaluation of Novel Digital Health Interventions

**DOI:** 10.2196/68757

**Published:** 2025-04-16

**Authors:** Catherine McCabe, Leona Connolly, Yuri Quintana, Arielle Weir, Anne Moen, Martin Ingvar, Margaret McCann, Carmel Doyle, Mary Hughes, Maria Brenner

**Affiliations:** 1 School of Nursing and Midwifery Trinity College Dublin Dublin Ireland; 2 Department of Clinical Informatics Beth Israel Deaconess Medical Centre Boston, MA United States; 3 Department of Medicine Harvard Medical School Boston, MA United States; 4 Institute for Health and Society, Faculty of Medicine University of Oslo Oslo Norway; 5 Department of Clinical Neuroscience Karolinska Institutet Stockholm Sweden; 6 School of Nursing, Midwifery and Health Systems University College Dublin Dublin Ireland

**Keywords:** digital health interventions, key performance indicators, Delphi technique, consensus methodology, drug-related side effects and adverse reactions, referral, consultation

## Abstract

Digital health interventions (DHIs) have the potential to improve health care and health promotion. However, there is a lack of guidance in the literature for the development, refinement, and prioritization of key performance indicators (KPIs) for the evaluation of DHIs. This paper presents a 4-stage process used in the Gravitate Health project based on stakeholder consultation and consensus for this purpose. The Gravitate Health consortium, which comprises private and public partners from across Europe and the United States, is developing innovative digital health solutions in the form of Federated Open-Source Platform and G-lens to present users with individualized digital information about their medicines. The first stage of this was the consultative process for the development of KPIs involving stakeholder (Gravitate Health project leads) consultations at the planning stages of the project. This resulted in the formation of an extensive list of KPIs organized into 7 categories. The second stage was conducting a scoping review, which confirmed the need for extensive stakeholder consultation in all stages of the KPI development, refinement, and prioritization process. The third stage was a period of further consultation with all consortium members, which resulted in the elimination of 1 category of KPIs. The fourth stage involved using the Delphi technique for refining and prioritizing the remaining 6 categories of KPIs. It is unusual to use this methodology in a nonresearch exercise, but it provided a clear consultative framework and structure that facilitated the achievement of consensus within a large consortium of 250 members on a substantial list of KPIs for the project. Consortium members ranked the relevance and importance of each KPI. The final list of KPIs provides substantial indicators sensitive to the needs of a broad group of stakeholders that are being used to capture real-world data in developing and evaluating DHIs.

## Introduction

Recent advancements in digital technology have enhanced many aspects of health care, allowing for digital monitoring, supporting health-related behaviors, and enabling communication [1]. One way this has been achieved is through the use of digital health interventions (DHIs), which have the potential to improve health care through illness prevention and health promotion [2,3]. DHIs are defined as a “discrete functionality of digital technology designed to improve health system processes in order to achieve health sector objectives” [4]. For example, DHIs may help facilitate tailored health solutions such as providing targeted support and education and enhancing treatment adherence [5] while reducing the burden on health care providers where time and resources are already limited [6]. However, evidence regarding the efficacy of DHIs to improve health outcomes has been mixed [7,8].

The Gravitate Health consortium comprises private and public partners from across Europe and the United States, drawing on expertise from within academia and research institutes, knowledge transfer experts, European Federation of Pharmaceutical Industries and Associations and the Innovative Medicines Initiative 2 partners, health care providers and payers, patient organizations and consumer groups, regulators and product information providers, and the digital technology industries. Gravitate Health is developing innovative solutions in the form of Federated Open-Source Platform and G-lens, which is an application, to present users with individualized digital information about their medicines, which is adapted to their personal needs and context. These solutions will access the electronic product information for each medicine that users are taking. It will use additional health data such as a personalized patient summary to focus and highlight the most personally relevant content from the electronic product information. The intended use of the Gravitate Health solutions is to enable users to understand their medicines in an individualized way, for example, understand why they are taking each medicine, what effects to anticipate, what precautions to take while consuming the medicine, and how best to follow recommendations and adhere to their ongoing treatment. It is anticipated that by being informed, users will better adhere to their medication schedule, which will lead to better health outcomes, fewer complications, and reduced health care use.

The Gravitate Health project work plan will see these solutions deployed and scaled across 4 demonstration sites using key performance indicators (KPIs) as a baseline for evaluation. KPIs are measures or variables that provide a means of observing and optimizing achievement and reflect changes connected to performance [9,10]. They deliver relevant information to stakeholders regarding the progress and sustainability of complex interventions [11]. One issue with selecting KPIs for the evaluation of innovative DHIs is that there is a lack of evidence for relevant performance indicators. This is because DHIs are by their very nature new and therefore have not previously been assessed. A recent scoping review by Brenner et al [12] concluded that there is a lack of evidence for a standardized approach to the development and prioritization of KPIs for DHIs. This paper highlighted that although most studies recognized and included the input of key stakeholders as a starting point for identifying potential KPIs, a common, rigorous process for the development and prioritization of KPIs for innovative DHIs is needed [12]. This paper addresses this lack of guidance within the literature for developing relevant KPIs for meaningful evaluation by presenting a novel use of the well-established Delphi technique as a process for refining and prioritizing KPIs for novel DHIs.

## Methods

### Process for Refinement and Prioritization of KPIs

Three potential approaches were considered as potentially suitable processes for refining and prioritizing the KPIs for evaluation in this project. The main criterion for consideration was the need for a process that would include all key stakeholders in coming to a consensus in refining and prioritizing KPIs. These were (1) the Sheffield elicitation framework [13], (2) a factorial survey approach [14], or (3) the Delphi technique [15]. The Sheffield elicitation framework and factorial survey approach rely on having a clearly defined group and a limited, strong evidence-based list of KPIs; therefore, neither were regarded as appropriate due to the uniqueness of the Gravitate Health solutions and the subsequent absence of supporting evidence. The list of KPIs for this project was produced at the outset of the project by key stakeholders based on their expertise or role in the project and reflected their aspirations for the digital solutions developed by the Gravitate Health project. Due to the novel nature of the solutions, the list of KPIs identified was lengthy and was not evidence-based. This meant that further refinement and prioritization of the KPIs was required at a more advanced stage of the project and needed to include all consortium members.

The Delphi technique was regarded as the most appropriate method to achieve consensus across all stakeholders in terms of refining and prioritizing the KPIs for evaluation of the Gravitate Health digital solutions. This technique, developed by Dalkey and Helmer [15], is a widely accepted process commonly used in research studies for converging opinions on a specific topic from experts within the discipline [16]. The Delphi technique is a systematic and interactive approach in research that involves a panel of experts coming together to share information and experience in order to achieve consensus as an outcome [17,18]. Consensus was an essential feature of refining and prioritizing the existing KPI list in the Gravitate Health project; therefore, using the Delphi technique as a consultative process rather than a research methodology provided a structured and rigorous framework to achieve this.

### Development of Initial KPIs and Evaluation

Based on the initial anticipated impact of the Gravitate Health digital solutions, a preliminary list of KPIs was created and classified according to anticipated areas of impact during a face-to-face informal brainstorming session by key stakeholders when the initial grant proposal was being prepared. This key stakeholder group comprised early consortium members representing medicine, patient representative groups, academia, technology, and pharmaceutical industry. The areas of expected impact of the KPIs included access and use, understanding, user experience, patient compliance or adherence, 2-way communication, and risk minimization. This classification of the KPIs and allocation of percentage thresholds for the achievement of these KPIs were based on expert opinion only and conducted using an informal consultative process at a very early stage in the proposal development. There was no specific evidence to support any other particular process for developing relevant KPIs in DHIs [12].

Measurement of KPIs throughout the project is an iterative process (3 intervals) using relevant tools at key stages throughout the project (minimal viable products 1, 2, and 3) to measure digital health literacy (of educational material), patient empowerment, user trust of the platform, usability, accessibility, and satisfaction with the platform. For example, systems data will be used to measure the use of the platform and to gauge user engagement. The Systems Usability Scale will be used to measure usability and the Medication Adherence Report Scale-10 to measure medication adherence [19,20]. Qualitative data will be collected via focus group interviews to obtain subjective accounts of user experiences related to trust, accessibility, usability, and user experience with digital solutions. The findings from each interval are used by the technical teams for product refinement and development throughout the project. Multimedia Appendix 1 describes the process of mapping KPIs to evaluation tools and evaluation time points.

### Stakeholder Consultation

Stakeholder consultation included a presentation of the initial KPIs at several internal meetings (monthly) within the Gravitate Health consortium, which had expanded in numbers significantly (n=250) since the initial list of KPIs was created. Each consortium member consulted during these meetings represented a key expert group involved in the ongoing design and evaluation of the Gravitate Health digital health solutions, for example, health care users, health care practitioners, academic institutes, and pharmaceutical and technology industries, and all were from high-income European Union countries. These meetings aimed to seek expert advice and perceptions on the initial KPIs and explain and discuss the use of the Delphi technique to refine and prioritize the KPIs. Overall, this consultative process demonstrated support for the preliminary list of KPIs with the exception of the category on “value for the industry and society.” These are specific deliverables for the project; therefore, it was perhaps not surprising that these KPIs were regarded as not applicable, as they would not be measurable by evaluation tasks in the project. This was an essential and supportive exercise in communicating the work of the consortium evaluation team to the broader stakeholder group and subsequently getting consensus on the process to be used.

### Delphi Survey and Consensus

**Figure 1:**
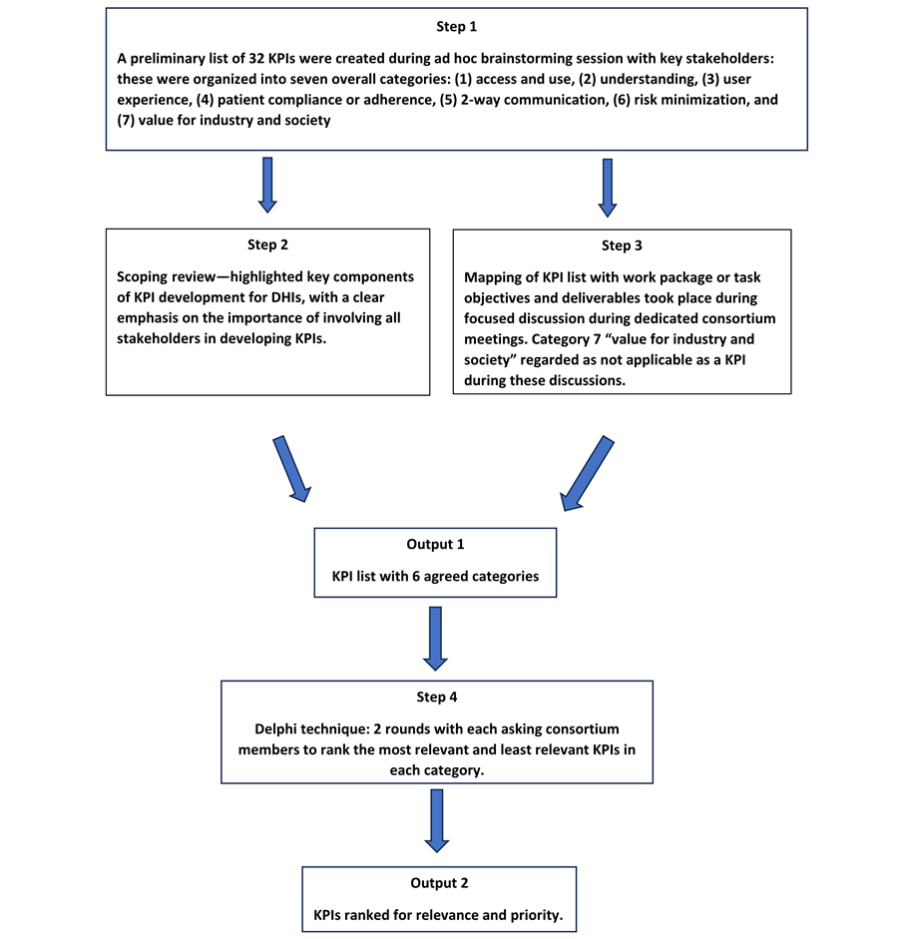
Flow diagram for consensus process for KPI development, refinement and prioritisation for evaluation of the Gravitate - Health project.

Value statements were created for each of the KPIs, and the list was revised to ensure that the wording was clear in terms of what the KPI was anticipated to measure in order to meet the SMART criteria defined in the World Health Organization guide for indicators: Specific, Measurable, Attainable, Relevant, and Time-Bound [21]. All members of the Gravitate Health consortium were then invited by email to contribute to the Delphi survey (n=250). The decision for inclusion of all members was made on consideration of the results from a previous scoping review, which highlighted a significant gap in the evidence-based knowledge in this domain, and strongly emphasized the involvement of stakeholders in developing KPIs for DHIs [12]. A 2-round ranking style Delphi was performed using the Qualtrics platform (version 082024) to identify and rank the final KPIs for use in the project (Figure 1) [22]. In round 1, all potential consortium members were sent a cover letter advising them of the purpose of the exercise. Survey items were presented as 2 questions for each of the 6 KPI categories. In the first question, consortium members were presented with the list of proposed KPIs for the category and asked to choose 1 KPI from the list they felt most represented the category and 1 KPI they felt least represented the category. This is in line with the commonly used and reliable approach of developing rank order in Delphi survey responses [23-25]. The second question was a free-text box asking consortium members if they would like to suggest an additional KPI that could be reflective of the category. In the first round of the survey, KPIs were listed in random order for each of the categories. Consortium members had 10 days to respond to round 1 and a further 10 days to respond to round 2 of the survey.

In round 2, consortium members were presented with the KPIs in ranked order based on results from the first round and again asked to rank the list in the order of most to least relevant. There were no differences in how consortium members ranked the KPIs in terms of relevance and priority in round 2. Minor wording edits were made during data analysis by study authors (MB and AW) to some of the KPIs in round 2 based on a small number of free-text feedback to increase the clarity and precision of the KPIs.

### Ethical Considerations

The conduct of this consultative process was solely intended for decision-making in relation to refining and prioritizing a predetermined list of KPIs. Therefore, ethics approval was not required. Participation in this process was voluntary, and participant engagement in this consultative process was regarded as consent. The Qualtrics platform was used to ensure all responses were anonymous. No compensation was given for engagement in the consultative process.

## Results

There were 40 respondents in the first round of the project and 41 respondents in the second round, which represents a response rate of 16%. As a research methodology, the Delphi technique has been criticized in health research for methodological issues such as low response rates [26]. However, as a process for consensus achievement in refining and prioritizing KPIs, the characteristics of the consortium members who responded in this process are representative of all key stakeholders in the Gravitate Health consortium. Just over 41% (n=17) from the pharmaceutical industry and 32% (n=13) from digital technology companies provided feedback in round 1 and 2. Other consortium members represented academic or research institutes (n=14, 34%; Table 1). Respondents listing their role as other (n=13, 32%) included the following: medical doctor, patient advocate, medicine agency, health professional working in academia, regulatory authority, user advisory group member, member organization in digital and telehealth, patient representative, and gerontologist or dementia specialist.

The results of the class ranks (the percentage of responses in which each KPI was listed as first in the order of importance) and mean ranks (average number of ranks for each KPI) for each of the 6 categories for the first and second Delphi rounds are presented in Multimedia Appendix 2.

The mean rank was calculated for each KPI within each category. These show the rankings for each KPI and the changes that occurred in the prioritization of individual KPIs between rounds 1 and 2. No additional KPIs emerged that were to be added to the list during this process. The overall final list of prioritized KPIs for the project is presented in Table 2.

**Table 1:** Characteristics of respondents.

Role	Round 1 (n=40), n (%)	Round 2 (n=41), n (%)
Industry	16 (40)	17 (42)
Academic or research institute	14 (35)	14 (34)
Health care providers	5 (13)	3 (7)
Patient representatives	6 (15)	5 (12)
Private partners	4 (10)	7 (17)
Digital technical expertise	14 (35)	13 (32)
Other	9 (23)	13 (32)

**Table 2:** Final list of refined and prioritized key performance indicators using consensus methodology.

Class rank			Key performance indicator
**Access and use**			
	1	Measure how usable and accessible the Gravitate Health platform is.	
	2	Measure citizens’ use of Gravitate Health to manage health information.	
	3	Measure if the platform is accessible on the web and personal devices.	
	4	Measure citizen awareness of the importance of self-management.	
	5	Measure European Union citizens’ awareness of Gravitate Health.	
**Understanding**			
	1	Measure if the Gravitate Health platform has educational material at a digital literacy level that is understandable.	
	2	Measure if the Gravitate Health platform provides users with an understanding of the medication benefits and how and why to take medication.	
	3	Measure if the Gravitate Health platform addresses physically, auditorily, or visually challenged users.	
	4	Measure if the Gravitate Health platform has the multilingual capability.	
	5	Measure if the Gravitate Health digital solution provides notifications and updates on prescription or over-the-counter electronic product information.	
	6	Measure if the Gravitate Health platform has reached the maturity of the technology platform at the end of the project.	
**User experience**			
	1	Measure if the Gravitate Health platform increases patient empowerment and activation.	
	2	Measure if the Gravitate Health platform facilitates patient empowerment and activation.	
	3	Measure if users trust the Gravitate Health platform.	
	4	Measure if the Gravitate Health platform improves the patient-provider interaction using G-lens.	
	5	Measure if the Gravitate Health platform is assessed by users as not providing information overload or missing relevant data.	
	6	Measure if the Gravitate Health platform provides user awareness through digital solution features.	
	7	Measure if the Gravitate Health platform provides caregivers (informal or nonprofessional) with a satisfactory experience.	
	8	Measure if the Gravitate Health platform provides health providers with a satisfactory experience.	
**Patient compliance or adherence**			
	1	Measure if the Gravitate Health platform leads to better medication compliance or adherence by users.	
	2	Measure if the Gravitate Health platform leads to better patient health outcomes.	
	3	Measure if the Gravitate Health platform leads to a safer use of medication or therapy administration.	
	4	Measure if the Gravitate Health platform leads to an increased partnership between the patient and the health team.	
**Two-way communication**			
	1	Measure if the Gravitate Health platform leads to increased provider awareness of own bias and understanding of users’ attitudes toward medication compliance or adherence.	
	2	Measure if the Gravitate Health platform will meet legal and privacy requirements while balancing a need to know, usability, and accessibility and will have a no-harm policy.	
	3	Measure if the Gravitate Health platform leads to an increase in patient opt-ins for providing real-world data.	
**Risk minimization**			
	1	Measure if the Gravitate Health platform leads to a risk minimization function.	
	2	Measure if the Gravitate Health platform provides alerts to users to avoid prescribed and over-the-counter drug interactions.	
	3	Measure if the Gravitate Health platform achieves lower risks across populations.	

## Discussion

### Principal Findings

Consortium members ranked the usability, accessibility of the G-lens, digital literacy, and patient empowerment as fundamental issues in this project. This reflects the drive for more accessible technology in health care, which had gained traction before the COVID-19 pandemic [27-29]. The most important KPI identified, risk minimization, relating to alerts for adverse interfering effects, is not surprising, given the previous work completed by the European Union’s Innovative Medicines Initiative WEB-RADR (Recognizing Adverse Drug Reactions), which highlighted the importance of detecting adverse effects early to improve patient safety [30]. However, the third-ranked most important item in this category was the need to achieve lower risks across populations. This reflects the importance of developing a health solution that enhances equity of care in medication management. Changes in KPIs were not observed during the refinement process in round 1 and 2 of the Delphi technique. This is not surprising, as the Gravitate Health digital solution did not exist prior to this exercise; therefore, consortium members had no prior experience of them.

### Comparison to Prior Work

The Delphi technique framework provides a complementary approach to the World Health Organization framework for monitoring and evaluating DHIs and to existing health system frameworks such as the Donabedian’s framework on quality of care, the Reach, Effectiveness, Adoption, Implementation, and Maintenance framework for public health, the logic model, and the balanced scorecard [21,31-34]. While these frameworks agree that setting indicators for planning and evaluation in DHI projects is important, they do not offer guidance on how this should be achieved. The process used for KPI development, refinement, and prioritization in the Gravitate Health project provides such guidance. It provides a standardized approach to achieving consensus between large groups of stakeholders and therefore can be used in many types of digital health solution projects in identifying and prioritizing KPIs.

The use of a Delphi technique in the Gravitate Health project provided a well-established framework for a structured approach to achieving consensus in developing, refining, and prioritizing KPIs in DHI initiatives involving large groups of diverse stakeholders where there is limited supporting evidence [12]. Current literature in this area shows that most previous initiatives for DHI evaluation used health assessment frameworks that were adapted for DHI evaluation focused on the usability and clinical outcomes of the interventions [12]. However, as Bradway et al [35] noted, new initiatives should be created with the unique properties of DHIs in mind. Though the specific KPIs identified by this project may not be generalizable to all DHI evaluations, this paper offers a useful first step toward a common methodology for the development of KPIs for DHIs.

### Limitations

This consultative process has some limitations. First, although the response rate was low (41 consortium members of 250 contributed), it has been noted that a minimum of 20 participants can achieve response stability in Delphi research studies [26]. The list of KPIs was mapped against feedback from health care users in the broader project; however, the project may have benefitted from wider engagement with a greater number of health care professionals, health care users, and health care organizations. This limitation is mitigated somewhat by all KPIs being measured regardless of the ranking, as the purpose of this project was to refine the initial KPI list and to develop an order of priority. For example, no additional KPIs were identified for inclusion. However, having a greater number of health care professionals and health care users may have led to a different outcome. In addition, limitations to this methodology include a limited number of items that can be included in a list, with some suggesting that the number of items be restricted to 3 to 5, with a maximum of 7, resulting in potential bias due to ties among items, response bias, and issues with standardization [22,36-39].

### Future Directions

Future work will conduct an external validation to measure the feasibility, reliability, or validity to demonstrate the robustness and applicability of the developed KPIs. This could be done by conducting more pilot testing of the indicators in real-world settings, assessing interrater reliability, and exploring the relationship between the KPIs and relevant outcomes.

### Conclusions

The objective of this paper was to present a consensus approach to refining and prioritizing candidate KPIs to measure the success of novel digital health solutions produced by the Gravitate Health project. The Delphi technique provided a structured framework for this, and albeit, a very novel context, it proved to be an effective approach. The final list of KPIs provides a list of substantial indicators sensitive to the needs of a broad group of stakeholders that can be used to capture real-world data in developing and evaluating DHIs. This consultative approach offers a suitable 4-stage process to enable the setting of evaluation indicators for the assessment of DHIs (Figure 1).

## Appendix

Multimedia Appendix 1 Process of mapping key performance indicators to evaluation tools and time points.

Multimedia Appendix 2 Class and mean rank for rounds 1 and 2 of key performance indicator refinement and prioritization.

## Abbreviations

DHI: digital health intervention
KPI: key performance indicator
RADR: Recognizing Adverse Drug Reactions

## Data Availability

The datasets generated or analyzed during this study are available from the corresponding author on reasonable request.
