# Supplementary material for: How to Refine and Prioritize Key Performance Indicators for Digital Health Interventions: Tutorial on Using Consensus Methodology to Enable Meaningful Evaluation of Novel Digital Health Interventions
Source: J Med Internet Res. 2025 Apr 16;27:e68757. doi: 10.2196/68757 (PMC12044311; doi:10.2196/68757)
Supplement: Multimedia Appendix 1 [file jmir_v27i1e68757_app1.docx]

| **Category** | **Key Performance Indicator** | **Evaluation tool** | **MVP1** | **MVP2** | **MVP3** |
| --- | --- | --- | --- | --- | --- |
| **Access and Use** |  |  |  |  |  |
| EU User awareness about the project | 10% annual increase in number of unique contacts globally | Systems data, platform/ website logs |  |  | ✓ |
| User awareness of importance of self-management | 15% annual increase of time on website/users, 25% increase in webinars attendance | Systems data, platform/ website logs |  |  | ✓ |
| Users use solution to manage health information | 85% of test users complete the pilot trial scenarios | Evaluation framework: Systems data, platform/ website logs | ✓ | ✓ | ✓ |
| Platform availability, usability, and accessibility | >50% of users evaluate *Gravitate health solutions* as useful, usable, and accessible | SUS, focus groups/ Interviews | ✓ | ✓ | ✓ |
| Platform availability and accessibility | Accessible platform available in web and personal devices | Adherence to WCAG 2.1 or GPII | ✓ | ✓ | ✓ |
| **Understanding** |  |  |  |  |  |
| Platform addresses physically, auditorily, visually, challenged, dyslexia requirements | 50% of physically challenged users’ express satisfaction with the digital services, above baseline | SUS and Qualitative data | ✓ | ✓ | ✓ |
| Multilingual capability | >5 languages supported | Selection of data |  | ✓ | ✓ |
| Digital literacy level of educational material | >75% information accessible for middle school level (9-year-old) | Formal tests in scenarios, SUS (system usability scale), focus groups/ interviews | ✓ | ✓ | ✓ |
| Maturity of the technology platform at end of project | TRL 4 to 8 for parts of the platform and digital solution |  |  |  | ✓ |
| Digital solution provides notifications and updates on prescription/OTC ePI | 10% annual increase in knowledge / use of platform features | Systems data, platform/ website logs, Online survey, interviews/ focus groups. |  |  | ✓ |
| Users understand medication benefits, how and why to take medication | 25% improvement over baseline according to content specific questionnaires | Focus groups/ Interviews | ✓ | ✓ | ✓ |
| **User experience** |  |  |  |  |  |
| Provider experience with user use of G-Lens | 25% baseline increase on platform impact on patients | Directed queries before during and after test. Online survey, focus groups/ interviews | ✓ | ✓ | ✓ |
| User empowerment and activation | 25% over baseline increase: self-Efficacy, motivation, activation (medium to strong effect) | PAM (Patient Activation Measure), engagement (measured by PHE-s). |  |  | ✓ |
| User empowerment through digital solution features | 25% increase in awareness of features, e.g. notifications, active search, interaction, queries | User satisfaction rating with service; Questionnaires; service logs Systems data, website and platform logs, SUS | ✓ | ✓ | ✓ |
| Patient empowerment and activation | 75% empowered to manage their medication, find relevant easily understood information on drugs, symptoms, and risks | Directed queries users before during and after test. Measures of psychological resilience, Health education impact Questionnaire (heiQ), individual interviews/ focus groups, PAM (Patient Activation Measure) |  |  | ✓ |
| Health provider satisfaction | 65% of providers report that digital *solution* has improved the patients’ treatment overview knowledge and health engagement | Self-reported satisfaction directed queries before during and after test. |  |  | ✓ |
| User assessment of G-Lens: no information overload or missing data | 60% satisfaction with digital solution features | Direct queries to all users before, after, during tests; use of the platform (system logs), individual interviews / focus groups, SUS | ✓ | ✓ | ✓ |
| Trust index for users | Medium to strong effect size on trust in health provider and health system, satisfaction doctor/patient relation/communication | HCCQ scale, Qualitative interviews/ focus groups |  |  | ✓ |
| Trust index for providers | Platform provides information needed | Direct queries to all users before, after, during tests, individual interviews / focus groups, |  |  |  |
| **User compliance/adherence** |  |  |  |  |  |
| Better medication compliance | Medium to strong effect size | Brief Medication Questionnaire, Hill-Bone Compliance Scale (Hill-Bone), 8-item Morisky Medication Adherence Scale (MMAS-8), Medication Adherence Questionnaire (MAQ) |  |  |  |
| Outcomes | 10% improved health outcomes effect size medium to strong | PROMs caught by portal for relevant targets |  |  |  |
| User Preferences / Co-creation | > 90% have tried the possibility of shaping cooperation with health team; > 50% use repeatedly | Quantitative interaction monitoring (sync/async) |  |  |  |
| safer use of medication/ therapy administration | 50% of users find that G-lens provides tailored information that helps them safely adhere to therapy in their lifestyle | Pediatric Quality of Life Inventory (PedsQL), health-related quality of life (scale SF12, HowRU, or 5WD) adherence, health literacy (MMOS-8; Hosborne scale), individual interviews/ focus groups |  |  |  |
| Two-way communication |  | HCCQ |  |  |  |
| Provider awareness of own bias and understanding of users’ attitude towards medication adherence | 25%-50% increase over baseline (Medium to strong effect size) on changing provider bias and improving understanding of users’ beliefs, fears and values on therapy adherence | Directed queries before during and after test, online survey, or Individual interviews / focus groups |  |  |  |
| Real world data | 10% annual increase patients opt in report/donate their data to the Gravitate-Health platform | Systems data, website, and platform logs |  |  |  |
| Legal and Privacy requirements balance “need to know”, usability, accessibility, and no harms policy | Accepted DPAA for all tests |  |  |  |  |
| **Risk minimization** |  |  |  |  |  |
| Risk Minimization Function | 85% providers receive alerts for adverse interfering effects and can prepare information about safe medication use | Decision tree signed off by expert group and appropriate medical agencies. Qualitative measures/ Systems data |  |  |  |
| Achieved lower risks across population | 50-75% confirmation of better user understanding of risks (Medium to strong effect size) | Purpose developed query, measure of health literacy (HLQ) |  |  |  |
| Alerts to avoid prescribed and OTC drug interactions | 20% of users over baseline take notice of this alert and discuss | Systems data and interview/ questionnaire data (combined). |  |  |  |
