# Supplementary material for: How to Refine and Prioritize Key Performance Indicators for Digital Health Interventions: Tutorial on Using Consensus Methodology to Enable Meaningful Evaluation of Novel Digital Health Interventions
Source: J Med Internet Res. 2025 Apr 16;27:e68757. doi: 10.2196/68757 (PMC12044311; doi:10.2196/68757)
Supplement: Multimedia Appendix 2 [file jmir_v27i1e68757_app2.pdf]

Category 1: Access and use					
Round 1			Round 2		
Class rank	KPI	Mean rank	Class rank	KPI	Mean rank
1	Measure citizens' use of Gravitate Health to manage health information - 85% of test users will complete the pilot trial scenarios.	2.38	1	Measure how usable and accessible the Gravitate Health platform is - Over 50% of users will evaluate Gravitate Health solutions as usable/ accessible.	2.02
2	Measure how usable and accessible the Gravitate Health platform is - Over 50% of users will evaluate Gravitate Health solutions as usable, /accessible.	2.25	2	Measure citizens' use of Gravitate Health to manage health information - 85% of test users will complete the pilot trial scenarios.	2.07
3	Measure if the platform is accessible on the web and personal devices - The platform is accessible on the web and personal devices.	3.48	3	Measure if the platform is accessible on the web and personal devices - The platform is accessible on the web and personal devices.	2.98
4	Measure citizen awareness of the importance of self-management - Citizens will receive a 15% annual increase in time on the website/users	3.28	4	Measure citizen awareness of the importance of self-management - There will be a 15% annual increase in time on the website by users.	3.69
5	Measure EU citizens' awareness of Gravitate Health - There will be a	3.62	5	Measure EU citizens' awareness of Gravitate Health - There will be a 10%	4.24

	10% annual increase in unique contacts globally.			annual increase in unique contacts globally.	
**Category 2: Understanding**					
Round 1			Round 2		
Class rank	KPI	Mean rank	Class rank	KPI	Mean rank
1	Measure if the Gravitate Health platform has educational material at a digital literacy level that is understandable - >75% of information is accessible to a middle school level 9-year-old.	2.88	1	Measure if the Gravitate Health platform has educational material at a digital literacy level that is understandable - >75% of information is understandable to a middle school level 9-year-old.	2.02
2	Measure if the Gravitate Health platform provides users with an understanding of the medication benefits and how and why to take medication - The Gravitate Health platform will achieve a 25% improvement over the baseline.	2.88	2	Measure if the Gravitate Health platform provides users with an understanding of the medication benefits and how and why to take medication - The Gravitate Health platform will achieve a 25% improvement over the baseline.	2.21
3	Measure if the Gravitate Health platform addresses users who are physically, auditorily, visually challenged, and have dyslexia requirements - 50% of physically challenged users will	3.08	3	Measure if the Gravitate Health platform addresses users who are physically, auditorily, and visually challenged - 50% of physically challenged users will express satisfaction with	3.52

	express satisfaction with the digital services above baseline.			the digital services above baseline.	
4	Measure if the Gravitate Health platform has the multilingual capability - The platform will have >5 languages supported.	3.10	4	Measure if the Gravitate Health platform has the multilingual capability - The platform will have >5 languages supported.	3.52
5	Measure if the Gravitate Health digital solution provides notifications and updates on prescription/ over-the-counter ePI - The Gravitate Health digital solution will achieve a 10% annual increase in knowledge/use of platform features.	4.50	5	Measure if the Gravitate Health digital solution provides notifications and updates on prescription/ over-the-counter ePI - The Gravitate Health digital solution will achieve a 10% annual increase in knowledge/use of platform features.	4.29
6	Measure if the Gravitate Health platform has reached the maturity of the technology platform at the end of the project - The platform will reach Technology Readiness Level 4 to 8 for parts of the platform and digital solution.	4.58	6	Measure if the Gravitate Health platform has reached the maturity of the technology platform at the end of the project - The platform will reach Technology Readiness Level 4 to 8 for parts of the platform and digital solution.	5.43
**Category 3: User experience**					
Round 1			Round 2		
Class rank	KPI	Mean rank	Class rank	KPI	Mean rank

1	Measure if the Gravitate Health platform increases patient empowerment and activation - The Gravitate Health platform will provide a 25% baseline increase in self-efficacy, motivation, and activation.	2.90	1	Measure if the Gravitate Health platform increases patient empowerment and activation - The Gravitate Health platform will provide a 25% baseline increase in self-efficacy, motivation, and activation.	2.64
2	Measure if the Gravitate Health platform facilitates patient empowerment and activation - The Gravitate Health platform will facilitate 75% of users with empowerment to manage their medication and find relevant, easily understood information on drugs, symptoms, and risks.	3.58	2	Measure if the Gravitate Health platform facilitates patient empowerment and activation - The Gravitate Health platform will facilitate 75% of users in managing their medication and facilitate finding relevant, easily understood information on drugs, symptoms, and risks.	2.88
3	Measure if users trust the Gravitate Health platform - There will be a medium to strong effect size on trust in health providers and the health system and satisfaction in doctor/patient relation/communication.	4.22	3	Measure if users trust the Gravitate Health platform - There will be a medium to strong effect on trust in health providers and the health system and satisfaction in doctor/patient relation/communication.	2.93
4	Measure if the Gravitate Health platform improves the patient-provider interaction using G- lens® -	3.85	4	Measure if the Gravitate Health platform improves the patient-provider interaction using G- lens® - The	4.57

	The Gravitate Health platform will provide a 25% baseline improvement in patient-provider interaction.			Gravitate Health platform will provide a 25% baseline improvement in patient-provider interaction.	
5	Measure if the Gravitate Health platform provides user empowerment through digital solution features - The Gravitate Health platform will provide a 25% increase in awareness of features (e.g., notifications, active search, interaction, and queries.	5.18	5	Measure if the Gravitate Health platform is assessed by users as not providing information overload or missing relevant data - 60% of users report satisfaction with the content in the Gravitate Health digital solution.	5.36
6	Measure if the Gravitate Health platform provides carers (informal/non-professional) with the satisfaction of the experience - 65% of carers report that the Gravitate Health digital solution has improved their knowledge and engagement with G-Lens.	5.30	6	Measure if the Gravitate Health platform provides user awareness through digital solution features - The Gravitate Health platform will provide a 25% increase in awareness of features (e.g., notifications, active search, interaction, queries.	5.93
7	Measure if users assess the Gravitate Health platform as providing no information overload or missing data - 60% of users report	5.45	7	Measure if the Gravitate Health platform provides carers (informal/non-professional) with a satisfactory experience - 65% of carers report a satisfactory	5.50

	satisfaction with digital solution features.			experience with the Gravitate Health digital solution.	
8R	Measure if the Gravitate Health platform provides satisfaction to health providers and is trusted - 65% of health providers report high satisfaction and trust with Gravitate Health digital solution.	5.33	8	Measure if the Gravitate Health platform provides health providers with a satisfactory experience - 65% of health providers report a satisfactory experience with the Gravitate Health digital solution.	6.19
**Category 4: Patient compliance and adherence**					
Round 1			Round 2		
Class rank	KPI	Mean rank	Class rank	KPI	Mean rank
1	Measure if the Gravitate Health platform leads to better patient outcomes - The Gravitate Health platform will lead to a 10% improvement in health outcomes.	2.02	1	Measure if the Gravitate Health platform leads to better medication compliance/adherence by users - The Gravitate Health platform will lead to a medium to strong effect size on medication compliance/adherence by users.	1.98
2	Measure if the Gravitate Health platform leads to better medication compliance/adherence by users - The Gravitate Health platform will lead to a medium to strong effect size on medication	2.22	2	Measure if the Gravitate Health platform leads to better patient health outcomes - The Gravitate Health platform will lead to a 10% improvement in health outcomes.	2.12

	compliance/adherence by users.				
3	Measure if the Gravitate Health platform leads to a safer use of medication/therapy administration - 50% of users will find that the Gravitate Health platform/ G- lens® provides tailored information that helps them safely adhere to therapy in their lifestyle.	2.22	3	Measure if the Gravitate Health platform leads to a safer use of medication/therapy administration - 50% of users will find that the Gravitate Health platform/ G- lens® provides tailored information that helps them safely adhere to therapy in their lifestyle.	2.60
4	Measure if the Gravitate Health platform leads to improved user preferences and co-creation - The Gravitate Health platform will lead to > 90% of users have tried the possibility of shaping cooperation with the health team, and > 50% will use it repeatedly.	3.52	4	Measure if the Gravitate Health platform leads to an increased partnership between the patient and the health team - The Gravitate Health platform will lead to > 90% of users participating in improved collaboration between the patient and the health team.	3.31
**Category 5: Two-way communication**					
Round 1			Round 2		
Class rank	KPI	Mean rank	Class rank	KPI	Mean rank
1	Measure if the Gravitate Health platform leads to increased provider awareness of bias and understanding of users’	1.73	1	Measure if the Gravitate Health platform leads to increased provider awareness of bias and understanding of users’ attitude towards	1.76

	attitude towards medication compliance/adherence - The Gravitate Health platform will lead to a 25%-50% increase over baseline on changing provider bias			medication compliance/adherence - The Gravitate Health platform will lead to a 25%-50% increase over baseline on changing provider bias	
2	Measure if the Gravitate Health platform will meet legal and privacy requirements while balancing "need to know," usability, and accessibility, and will have a no-harm policy - Data Processing Agreement will accept the Gravitate Health platform for all tests.	1.98	2	Measure if the Gravitate Health platform will meet legal and privacy requirements while balancing a need to know, usability, and accessibility, and will have a no-harm policy - Data Processing Agreement will accept the Gravitate Health platform for all tests.	1.83
3	Measure if the Gravitate Health platform leads to an increase in patient opt-ins for providing real-world data - The Gravitate Health platform will lead to a 10% increase in users who opt into a report/donate their data to the Gravitate-Health platform.	2.30	3	Measure if the Gravitate Health platform leads to an increase in patient opt-ins for providing real-world data - The Gravitate Health platform will lead to a 10% increase in users who opt into a report/donate their data to the Gravitate-Health platform.	2.40
**Category 6: Risk minimization**					
Round 1			Round 2		
Class rank	KPI	Mean rank	Class rank	KPI	Mean rank

1	Measure if the Gravitate Health platform leads to a risk minimization function - 85% of providers will receive alerts for adverse interfering effects and can prepare information about safe medication use.	1.95	1	Measure if the Gravitate Health platform leads to a risk minimization function - 85% of providers will receive alerts for adverse interfering effects and can prepare information about safe medication use.	1.83
2	Measure if the Gravitate Health platform achieves lower risks across populations - The Gravitate Health platform will achieve a 50-75% confirmation of better user understanding of risks.	1.98	2	Measure if the Gravitate Health platform provides alerts to users to avoid prescribed and over-the-counter drug interactions - The Gravitate Health platform will lead to a 20% increase over the baseline of users taking notice of alerts to avoid prescribed and over-the-counter drug interactions.	1.98
